# “*Tobacco Is the Chief Medicinal Plant in My Work*”: Therapeutic Uses of Tobacco in Peruvian Amazonian Medicine Exemplified by the Work of a *Maestro Tabaquero*

**DOI:** 10.3389/fphar.2020.594591

**Published:** 2020-10-07

**Authors:** Ilana Berlowitz, Ernesto García Torres, Heinrich Walt, Ursula Wolf, Caroline Maake, Chantal Martin-Soelch

**Affiliations:** ^1^Unit of Clinical and Health Psychology, Department of Psychology, University of Fribourg, Fribourg, Switzerland; ^2^Institute of Anatomy, Faculty of Medicine, University of Zurich, Zurich, Switzerland; ^3^Independent Practitioner, Loreto, Peru; ^4^Department of Oral- and Cranio-Maxillo-Facial Surgery, University Hospital Zurich, Zurich, Switzerland; ^5^Institute of Complementary and Integrative Medicine, Faculty of Medicine, University of Bern, Bern, Switzerland

**Keywords:** tobacco, *Nicotiana rustica* L., Amazon, medicinal plant, Peru, psychoactive, psychedelic-assisted therapy, traditional medicine (TM)

## Abstract

**Introduction:**

Harmful usage of tobacco is a public health problem of global concern and, in many countries, the main risk factor for non-communicable diseases. Yet, in the Peruvian Amazon, the geographical region believed to be tobacco’s historical birthplace, this plant is associated with a strikingly different usage and repute: Tobacco (especially *Nicotiana rustica* L.) in this area is described as a potent medicinal plant, used topically or *via* ingestion to treat a variety of health conditions. The goal of this transdisciplinary field study was to investigate clinical applications of the tobacco plant as per Amazonian medicine exemplified in the practice of a reputed *Maestro Tabaquero*, an Amazonian traditional healer whose medical specialization focuses on tobacco-based treatments.

**Methods:**

Using a transdisciplinary clinical approach, we conducted in-depth interviews with the tabaquero applying the systematizing expert interview method, in order to map modes of preparation and administration, indications, contraindications, effects, risks, adverse effects, and systemic aspects of tobacco-based remedies.

**Results:**

The informant’s descriptions revealed refined knowledge on this plant’s therapeutic properties and scope, safety profile, and application techniques. The main indications mentioned included “problems of the mind,” of the respiratory system, parasitic illnesses (intestinal/skin), gout, and Amazonian epistemic conditions described as spiritual-energetic in nature. A liquid remedy taken orally was his most commonly used preparation, with acute/sub-acute effects involving a pronounced psychoactive component (altered state of consciousness) and physiological response (emesis, nausea). A skilled tabaquero that knows how to dose, administer, and intervene in case of adverse effects was considered imperative for safe treatment delivery.

**Conclusions:**

To our knowledge, this is the first study employing a transdisciplinary clinical approach to examine therapeutic applications of tobacco by an Amazonian tabaquero. Our findings significantly contribute to the growing research literature on Amazonian medicine and emergent psychedelic-assisted therapies and could, in the long-term, open new treatment avenues in several domains. Forthcoming studies should assess toxicity/safety and clinical outcomes of patients receiving Amazonian tobacco-based treatment.

## Introduction

Harmful usage of tobacco (*Nicotiana* spp.) is considered a public health problem of global concern, with 1.1 billion smokers worldwide (World Health Organization, 2017). In many countries, it is the main risk factor for non-communicable diseases (Forouzanfar et al., 2015). Yet, in the Amazon, the geographical region believed to be tobacco’s historical birthplace (Oyuela-Caycedo and Kawa, 2015), this plant, especially *Nicotiana rustica* L., is associated with a strikingly different usage and repute (Russell, 2019): Tobacco is described as the main curing tool of Amazonian healers (Barbira-Freedman, 2015) and generally portrayed as a “Master Plant” in this context (Russell and Rahman, 2015). The ethnographic literature describes tobacco uses for therapeutic purposes in conjunction with millennia-old Amerindian traditions (Wilbert, 1993) and highlights the central role the plant plays particularly in Amazonian medicine. For the Amazonian natives, the link between medicine and tobacco is so basic, that the generic term for “healer” in several local languages is etymologically linked to the word for “tobacco,” for instance for the *Yuracaré* people, where *korrë-n-chata* (i.e., healer) literally means “he who eats tobacco” (Thomas et al., 2011). Similarly for the *Ashaninka* (Russell and Rahman, 2015) and the *Matsigenga* (Shepard, 1998) people, whose words for “healer” (*sheripiari* and *seripigari*, respectively) literally translate to “the one intoxicated by tobacco.” The Amazonian *Keshwa* people describe tobacco as the “father of all plants” (Barbira-Freedman, 2010; Barbira-Freedman, 2015). The latter description is not unlike informal accounts from our own previous fieldwork where tobacco was referenced as the “director of all medicinal plants.” In light of these descriptions, it seems striking that this plant in the rest of the world is used as a recreational drug, with tobacco smoking being the most prevalent addictive behavior across the planet (Martin-Soelch, 2013; Gowing et al., 2015).

Tobacco’s historical migration from indigenous Latin America and its transformation from local medicinal plant to global substance of abuse ensued *via* its rapid popularization among Spaniards after the colonial arrival on the American continent: The plant was introduced to Europe in the late 15th century, where it was readily adopted, and an extensive chain of trade later stretching to Africa and Asia took its course (Oyuela-Caycedo and Kawa, 2015). Notwithstanding an initial interest in tobacco’s medicinal possibilities among the new users (Charlton, 2004), tobacco’s large-scale commodification and global spread brought forth novel consumption patterns, recreational uses, which—in stark contrast to the plant’s native origins in indigenous healing—turned out to have deleterious health outcomes, as today is well known (Russell and Rahman, 2015; West, 2017). That the plant has traveled to the West without the corresponding indigenous know-how on its medical uses could be hypothesized to have played a role in these developments.

Traditional medicines, defined as “knowledge, skill and practices based on the theories, beliefs and experiences indigenous to different cultures, whether explicable or not, used in the maintenance of health, as well as in the prevention, diagnosis, improvement or treatment of physical and mental illnesses” (p.8, WHO, 2019), play an increasingly central role in global modern healthcare, particularly for chronic conditions like cancer, diabetes, or chronic pain (Molassiotis et al., 2005; Turk et al., 2011; Ablin et al., 2013; WHO, 2013; Falci et al., 2016; Charlier et al., 2017). The WHO (2019) considers its inclusion to global primary healthcare as pivotal for achieving their declared goal of Universal Health Coverage and the Sustainable Development Goals (United Nations, 2015). This is particularly relevant also for the psychosomatic and mental health domain (Gureje et al., 2015), where treatments are known to be markedly underprovided on a global scale (see worldwide treatment gap in mental health; Kohn et al., 2004; WHO, 2008; Evans-Lacko et al., 2017) and where chronicity is common (Costello et al., 2002; Brandon et al., 2007; Koob and Le Moal, 2008; WHO, 2008). Among traditional medical systems currently investigated for mental health, especially the Amazonian tradition and its psychoactive ayahuasca brew (*Banisteriopsis caapi* and varying admixture plants; Riba et al., 2003; McKenna, 2004) have recently become the center of scientific attention across the globe (Feeney et al., 2018; Hamill et al., 2018; Labate and Cavnar, 2018). Clinical research in this context has been burgeoning over the last years, alongside research on other psychoactive substances (see also “psychedelic-assisted therapies”; Tupper et al., 2015; Johnson et al., 2019), with growing evidence showing ayahuasca to benefit affective disorders, substance use, anxiety disorders, and other conditions (Thomas et al., 2013; Labate and Cavnar, 2014; Osorio Fde et al., 2015; dos Santos et al., 2016; Nunes et al., 2016; Coe and McKenna, 2017; Domínguez-Clavé et al., 2018; Palhano-Fontes et al., 2018; Renelli et al., 2018; Berlowitz et al., 2019). However, notwithstanding the considerable global interest in ayahuasca and Amazonian medicine, the nature and place of tobacco-based treatments within this medical system have so far been neglected: To our knowledge, no clinical research on Amazonian medicine has yet systematically examined these uses—in spite of the central role the anthropological literature ascribes to this plant in Amazonian healing (Valadeau et al., 2010; Barbira-Freedman, 2015). Also in a more general sense, even though tobacco as medicine and mind-altering substance is ubiquitously found in ethnographic accounts of Amerindian societies, its therapeutic uses have received remarkably little scholarly attention thus far (Russell and Rahman, 2015).

The overall goal of the current study was to document in detail how the tobacco plant is used in Peruvian Amazonian medicine as exemplified in the practice of an Amazonian specialist or *Maestro Tabaquero*, a reputed Amazonian healer whose medical specialization focuses on tobacco uses (Valadeau et al., 2010). More specifically, performing in-depth interviews in the scope of a transdisciplinary collaboration (Jahn et al., 2012), we aimed to report indications, contraindications, modes of preparation and application, short- and long-term effects, risks, and adverse effects of tobacco-based treatments; we further aimed to understand the contextual or systemic aspects of how this plant relates to other treatment constituents of Amazonian medicine as a system.

## Methods

The systematizing expert interview method (Bogner and Menz, 2009) was employed to collect in-depth information on the above topics using a single-respondent key informant design (Tremblay, 1957; Sandelowski, 1996; Meuser and Nagel, 2009a). An expert is defined as a person who, due to her/his experience or position, is in the possession of special knowledge regarding a socially relevant field; the expert interview, then, is a method for gaining access to this special knowledge or expertise (Gläser and Laudel, 2010). This interviewing method requires the interviewer to be knowledgeable in the subject at hand (Meuser and Nagel, 2009b), otherwise the interviewee may avoid complex terrain (expecting the interviewer to be insufficiently competent to understand it) and share only superficial information. Given the multi-epistemic context of this study, an interviewer who is a relevant specialist from a different knowledge culture (Bogner and Menz, 2009)—namely a scientist in clinical and health psychology with experience in investigating Amazonian medicine (first author of this paper; e.g., Berlowitz et al., 2017; Berlowitz et al., 2019)—was considered most suitable. This allowed equitable dialogue and in-depth exploration of knowledge, including aspects that differ in the two epistemic domains.

Given the scarcity of literature presently available on the subject and thus exploratory nature of this work, and in light of this study’s clinical focus as opposed to anthropological (i.e., of interest is clinical depth rather than cultural representativeness), a single-respondent design involving continual sittings over an extended period of time was chosen in favor of a multiple respondents design with one sitting each (Etkin, 1993; Sandelowski, 1996; Bengtsson, 2016). A high level of information power (Malterud et al., 2016) was nonetheless safeguarded by selecting a highly accomplished and specialized clinical expert as the key informant of this study (Gläser and Laudel, 2010; Krause et al., 2018).

### Instrument

A comprehensive semi-structured interview schedule was developed *via* a methodological procedure described by Kallio et al. (2016): We generated an ample set of guiding questions to cover the different content areas of the research subject, drawing from ethno-medical field research and classification methods (Lipp, 1989; Staub et al., 2015), our previous research experience and the anthropological literature on Amazonian medicine and tobacco uses (Wilbert, 1993; Russell and Rahman, 2015; Berlowitz, 2017), as well as general psychological/somatic symptom category systems (Schinka, 1989; Derogatis and Unger, 2010). The resulting inventory was handed to an interdisciplinary expert panel for assessment consisting of scientific advisors from medicine, psychology, complementary medicine, biology, and medical anthropology, and was adapted accordingly. The semi-structured format was chosen due to the open, co-creative ambiance it generates in the interviewing process (new topics and follow-up questions may be improvised by both parties), which in turn increases breadth and depths of material (deMarrais and Lapan, 2003). The final interview schedule consisted of nine chapters (see **Table 1**), each made of sub-chapters with several probing questions. The full schedule can be obtained from the authors upon request.

**Table 1:** Chapters and subchapters of the interview schedule.

Part 1: Training in Amazonian medicine/Tabaquerismo and details of informant 1.1. Training in traditional Amazonian medicine and specialization in tobacco 1.2. Basic sociodemographic data
Part 2: Systemic aspects 2.1. The *Tabaquero/a* as Amazonian specialist in the medical usage of tobacco 2.2. Tobacco in relation to the rest of the traditional Amazonian medical system
Part 3: Modes of preparation and manners of application 3.1. Liquid preparation for ingestion 3.2. Manners of application of liquid tobacco 3.3. Solid preparation for ingestion 3.4. Topical application 3.5. Smoked form
Part 4: Short- and long-term effects of ingested liquid tobacco 4.1. Physical effect profile 4.2. Emotional effect profile 4.3. Cognitive effect profile 4.4. Other domains of effects 4.5. Dose-dependent effects
Part 5: Indications for tobacco-based medicines 5.1. Mental health domain 5.2. Amazonian epistemic domain 5.3. Physical health domain 5.4. Other health-related domains
Part 6: Contraindications for tobacco-based medicines for ingestion 6.1. Physical 6.2. Psychological 6.3. Specific life circumstances
Part 7: Diagnostic procedures and treatment choice 7.1. Determination of indicated type of medicine
Part 8: Risks, adverse effects, and interactions 8.1. Adverse effects 8.2. Risks 8.3. Interactions with other substances
Part 9: Broader perspective and final considerations 9.1. Broader perspective on tobacco uses 9.2. Final considerations

Each subchapter consisted of a number of questions and probes in the full interview schedule.

### Key Informant

The informant was a 51-year-old Amazonian traditional healer (*curandero*) and Maestro Tabaquero with 36 years of clinical experience. He is from Peru, but of mixed Amazonian ancestry, as his four grandparents were originally from Peruvian, Colombian, Ecuadorian, and Brazilian Amazonian areas, respectively. Born at the banks of the Peruvian Río Tigre (Loreto Province), the informant was raised by his maternal grandparents who were traditional healers and farmers. He was instructed by his grandparents from an early age on and started to train more formally at age 8, *via* extended forest retreats (*dietas*; solitary retreats designed to experientially study medicinal plants), as is customary in the training of an Amazonian healer. At age 12 he discovered his calling to specialize as a tabaquero, after having been initiated into the tobacco medicine by his Peruvian grandfather. He recalls starting to regularly attend to patients at age 15, but he continued to develop his knowledge and skill in the field of healing and tobacco-based treatments throughout his life, also including periods of study with Amazonian healers other than his grandparents. He progressively attained expert status in the field of tobacco medicine and the reputation of a *Maestro*, a highly esteemed Amazonian healer. Today he attends regional, national, as well as international patients at his practice in Loreto, offering treatments using tobacco-based medicines in the scope of one-time consultations, individual retreat-like treatments, and occasionally also group work.

### Data Collection Procedure

A total of eight interview sessions were held between October 2019 and July 2020 after written informed consent was obtained from the informant. Interviews were conducted face to face at the informant’s workplace in Loreto (Peru) except for the last session, which was conducted long-distance (over the phone) due to the COVID-19 pandemic. All sessions were held in Spanish (native language of the informant). Interviewing was repeated as long as relevant new materials continued to emerge, and was discontinued when the level of depth reached was considered satisfactory (depth defined in terms of detail and completeness, Weiss, 1994; deMarrais and Lapan, 2003, akin to the concept of saturation, Morse, 1995; Saunders et al., 2018). In order to facilitate subsequent transcription of the material all interview sessions were audio-recorded, with the informant’s permission. The study was approved by the ethics committee (2019; Ref-No.:88A1; Fribourg) and conducted in accordance with international regulations and the Declaration of Helsinki.

### Data Analysis

The audio-recorded interview sessions were transcribed verbatim and analyzed using a manifest content analytic approach, aimed to condense the rich information conveyed by the informant and systematize it relative to the main interview questions (Graneheim and Lundman, 2004; Mayring, 2008; Bengtsson, 2016). A preliminary category system made of seven themes (see *Results*) was initially developed based on the interview questions and adaptations during the data collection process. The textual material was carefully analyzed and structured along those themes in a first analytic step. Additional relevant themes that emerged during this process were added to the category system inductively in a second step; additionally, if the material suggested so, adjustments were made to the foci of predefined themes (e.g., narrowing, broadening, further differentiating, merging, or splitting multifaceted clusters). Once the final category system consisting of nine themes (see next section) was established, the overall material was classified again using this category system in a final step.

## Results

The themes initially defined based on the interview questions were (in slightly adapted form): Manners of preparation and modes of application; Experienced short-, mid-, and long-term effects; Applications in the physical health domain; Applications in the mental health and psychosomatic domain; The tobacco’s spiritual-energetic properties and applications in Amazonian epistemic health domains; Contraindications, interactions, risks, and adverse effects; and Tobacco in relation to other Amazonian medicinal plants. The first two themes mentioned were split into two distinct categories in the course of the analytic process, and an emergent theme (Uses of tobacco smoke in the work of a Tabaquero and Amazonian medicine) was added. For each theme, a condensed summary of contents is presented, along with quotes to exemplify or illustrate the material in the words of the healer. Some details provided by the informant were omitted from the report in order to protect the intellectual property of traditional knowledge by this healer and the Amazonian medical tradition overall (see also Nagoya protocol, Buck and Hamilton, 2011). Especially precise recipes are not provided here, so as to protect the intellectual property of traditional knowledge. Moreover, the Tabaquero also expressed concern that if recipes were to be made public, readers could get tempted to experiment with tobacco on themselves or others, which would be highly dangerous.

### Therapeutic Uses of the Tobacco Plant: Manners of Preparation and Routes of Administration

The healer explains that he relies primarily on leaves of *N. rustica* L. for preparing his remedies, but that other parts of the plant are also useful for certain problems (e.g., the root applied on dogs for skin worms). “The tobacco [I use] is prepared of leaves, it is called cured, it has a process of curing the leaves, and once they have a color between black and brown, they are ready to be used.” He reports making liquid and solid remedies from this tobacco, some for ingestion, others for topical application (see **Table 2**). The Tabaquero reports using many “varieties of preparations and combinations of medicinal plants” in conjunction with tobacco, depending on the type of illness the remedy is supposed to target. Liquid preparations for ingestion are what he uses most frequently. Unless otherwise stated, the following sections will therefore focus on this type of remedy.

**Table 2:** Types of tobacco-based preparations and routes of administration described by the informant.

Preparation: Route of administration:	Liquid	Solid	Smoke-form
Topical	Medicated shower with tobacco leaves	Cataplasms or salve/paste, applied on affected area	*Soplada* on defined energy points of patient body (akin to acupoints)
Oral	Ingestion; most commonly used method by informant	*Tabaqueros* only (not used for patients)	*Tabaqueros* only (smoke swallowed rather than inhaled)
Intranasal	Liquid snuff; often combined with oral route	Pulverized tobacco snuff (not used by informant)	(some passive nasal inhalation may occur during *sopladas*)
Rectal	Tobacco-based enema (not used by informant)	–	–

The healer uses oral, nasal, or topical routes of administration for his tobacco remedies. In case of the nasal route he uses liquid, but not solid tobacco snuff, as he considers the latter (today often called *rapé*) to have certain disadvantages. For topical application he uses tobacco leaves, either as a cataplasm or processed with other plants as an ointment or in the form of a medicated shower (“baño de plantas”). He does not employ enemas of tobacco, but states that this is a common practice in the Amazon and may be useful for instance for intestinal parasites. Finally, he makes use of the leaves in smoked form for “energetic purposes,” as he explains, a concept which will be further described in forthcoming sections.

### Modes of Application: Dosage and Ritual Context

Dosage is determined as a function of type of illness and constitution of a person’s body, nervous system, and energy, the healer explains. An oral tobacco dose can range from minuscule to large, and a thorough diagnostic analysis beforehand is critical, the healer asserts: A tabaquero must learn the traditional Amazonian techniques (e.g., *pulsar*, see also *Contraindications, Interactions, Risks, and Adverse Effects*) for this purpose during his training, explains the healer. He must also learn the Amazonian ritual techniques for correctly administering the medicine (e.g., *soplar*, *icarar*, see *Uses of Tobacco Smoke in the Work of a Tabaquero and Amazonian Medicine*), and the methods for intervening in case of adverse reactions to the tobacco effect (see *Contraindications, Interactions, Risks, and Adverse Effects*). He portrays these methods as a pivotal part of the safety protocol for using medicinal tobacco securely.

The healer describes several different contexts for administering oral tobacco, depending on the type of preparation and illness. For “strong preparations” he conducts tobacco ceremonies, which can be held for an individual or in a group setting, or he administers the tobacco in the scope of Amazonian dietary retreats (traditionally called *dieta*). The latter involves a period of several days of social seclusion, strict dietary regime, and daily tobacco ingestion. In yet other cases he may prescribe a tobacco preparation combined with further medicinal plants that target specific health problems. For all of these three contexts, however, a specific dietary regime is mandatory: On the days of tobacco ingestion the regime is strict, and for several days to weeks after the treatment, specific foods and behaviors need to be avoided. “Also, one has to know how to eat,” the healer adds, implying that an overall healthy diet during this timeframe is important.

### Experienced Short-, Mid-, and Long-Term Effects

The healer states that the felt effect of tobacco heavily depends on the specific mode of preparation. He goes on to describe the sequence of effects typically experienced in the context of the “strong” tobacco preparation used in the ceremonial or dietary context: The first effects may set in quite immediately or after a few minutes, the onset being experienced either abruptly (“an internal blow”), or more gradually as an intensifying malaise. The patient may experience an inner tension with typical bodily sensations (dizziness, nausea, weakness) and cognitive/affective unease, a process which then culminates in vomiting. This tobacco-induced emesis gives way to a next effect phase, in which an inner sense of relaxation, coupled with introspective mental activity, tends to be experienced. The healer describes: “About 30 minutes after having ingested the tobacco, after having vomited, there is an opening of the psychic-mental. The person starts to reflect and there are like stories surfacing, possibly from childhood. This is because the psychic-mental has opened—a bit like a memory chip of a cell phone where there is information. And there the person will see or feel a lot of bad things, and a lot of good things. These things are not perceptions of something external, but they are emerging from their own mind, it is their mind that is releasing or liberating things. These are the effects. And later on, if the body and the tobacco have aligned, the person feels a sort of inner peace. It has this effect.” He describes that memories, emotions, and thoughts are surfacing in this phase and “if someone knows a bit of concentration, there may also be visions.” This progression of effects may take approximately 2 h, he explains, after which the intense effect wanes but remains in a mild form. Vivid dreams are common in the sleep thereafter, the healer explains, often with personally meaningful contents.

He describes that in the days or week that follow, one typically discovers a more positive outlook on life, a more joyful general attitude. Prior negative tendencies of the mind have greatly lessened and “a positive mind-state gets strengthened.” On an emotional level, the healer describes that “if for instance someone has been very hard in their heart, he/she will have feelings come up. The person may experience more love, love towards nature, animals, or people. It opens a state of feeling which may be new to that person; they may be happy, or cry for example.”

The nature of long-term therapeutic benefits of tobacco-based remedies generally depends on the specific illness targeted (see the following 3 sections), but if administered for cleansing purposes, an occasional repetition is necessary, to prevent new accumulations of toxins in the body-mind system, so to speak: “So, since the contemporary world is quite contaminated, in different ways, it is necessary to continually liberate and clean these energies. [… ] Like a machine that needs regular maintenance, something like that.”

### Applications in the Physical Health Domain

The healer lists problems of the respiratory system like sinusitis, gout, parasitic illnesses of intestines, and epilepsy as the main types of physical health problems that he treats with tobacco. He specifies that “there is a process for preparing the tobacco which makes it into effective medicine for the lungs. In other uses it may become a poison for the lungs, but if it is processed in the right manner, it is medicine, it cures; the tobacco absorbs phlegm and removes it [from the respiratory system].” He goes on to describe a different manner of preparation, which he uses for treating intestinal parasites; a remedy that “is like a purge, it gives you diarrhea, it cleans [the intestines].” He explains that in general, tobacco is a hot or heating herb. It is therefore apt to treat inner cold conditions, but not suited for conditions associated with inner heat. Gases in the intestines that arise due to excessive cold, he exemplifies, can effectively be treated with tobacco; yet tobacco is not advised for an acute inflammation in the intestines.

The healer points to the interplay between the physical and psychological domains in this context. He describes the existence of a direct connection between intestines and brain, with a chain reaction occurring on account of this linkage when drinking tobacco: “Physically it [the tobacco] cleans the intestines of parasites and negative energies, but because the intestines connect to the brain, if the intestines get liberated, the brain gets liberated as well.” This manifests as improvements to psychological well-being (see next section). He explains that similarly, when mucus and phlegm that block the respiratory pathways get expelled with the help of tobacco, there is an increase of oxygen flow which leads to an “opening of the brain” and an easing or clearing of the mind: “For many foreigners that are [psychologically] unwell, it is because the brain is closed off of oxygen. Only very little can pass and that is why they feel bad. This is why the tobacco helps, when it makes you vomit and spit out the phlegm, the phlegm that gets removed unblocks the pathways here, and here [pointing to specific spots on the body], and then the person sees things differently.” Another type of tobacco preparation is effective for gout, he continues, a condition he relates to inner cold. Further, topical application of a tobacco ointment is helpful for parasites of the skin. A specific preparation of tobacco combined with other ingredients is used to treat epilepsy. Finally, the healer adds that tobacco has benefits also for a generally healthy person, because it fortifies and detoxifies the body. This is why traditionally it is employed also as a preventative or maintenance mechanism to prevent future illness.

Taking the concept of physical health domain in a broader sense (i.e., beyond human anatomy), the healer describes using tobacco also in veterinary medicine, especially for dogs, for instance for parasitic illnesses (skin or intestinal), but it can also be administered to enhance their olfactory sense: “In case of the dog, [the tobacco] is applied in his nose. In the nose because it makes him smell other animals from a large distance. The dog becomes an excellent hunter.” The healer adds that a similar effect, i.e., an increase in sensory acuity also happens for humans that ingest tobacco, but that they may often not notice it. Finally, he adds that tobacco can be used for the health of crops, for instance if a plant gets infected with a pest, tobacco may be sprayed on it.

### Applications in the Mental Health and Psychosomatic Domain

The healer continually describes that the tobacco remedy “centers” or “strengthens” the mind. Correspondingly, among the chief indication for this medicine he points to “problems of the mind” or of the “psychic-mental system.” According to his descriptions this can include clinical or subclinical mental health issues, but excludes certain severe psychiatric conditions, for which tobacco is contraindicated. He describes therapeutic benefits for psychological processes related to attention (“some people think, and later they forget; others don’t think at all—these things the tobacco helps to clear, or open”), cognitive tendencies (“if previously a person had a destructive mind, [after the tobacco-treatment] they don’t have it anymore, the mind is more constructive, there is a change”), mood and self-image (“a person with a very low self-esteem, if they drink tobacco the self-esteem gets lifted, the tobacco can help”; “the person feels more cheerful, more serene”), as well as fearfulness (“[the tobacco] strengthens the mind, the brain—if previously seeing a cockroach made me react with fear, now the cockroach is my pet, as an example”).

The healer describes a tonifying remedy that targets depression or anxiety conditions specifically. Severe clinical cases may require treatment between 3 and 6 months, but 1 month of treatment with this remedy is generally sufficient for milder cases. However, the Tabaquero explains that for certain types of clients with a chronic nervousness (“nervios crónicos”) or an overwrought nervous system (“sistema nervioso muy afectado”), tobacco is contraindicated. He explains that for such patients, even though their body would be apt to a tobacco-treatment, there is a potential that their mind reacts to the tobacco effect with panic or loss of control. Tobacco is also contraindicated for severe psychotic conditions. In less severe cases (e.g., mild forms of paranoia), the healer explains that a plant combination that includes tobacco can help, but that in most cases, a more structured treatment frame (also involving counseling) is necessary. Similarly, for highly aggressive or hostile patients tobacco is contraindicated. The healer illustrates that for pathologies that feature excessive heat in the head, such as the former, tobacco may in fact aggravate the condition, due to the heating quality of the herb. Further, in the context of substance use disorders, the healer expresses that although tobacco can help in principle, a more extensive overall treatment frame is needed, and that for such patients tobacco can only be prescribed if they are already abstinent: “They are generally compromised and their brain affected, requiring another kind of therapy to stop using. After about 6 months without using the drug, then they can however drink tobacco,” which will help the detoxification of body and energy, the healer explains, since the pathology involves both “physical and energetic ills, and the tobacco cleans these things.” He points out that for alcoholics, conversely, repeated tobacco ingestion can be used to help them achieve abstinence. This is also the case for cigarette smoking cessation: “If the person drinks tobacco several times over an extended period of time, then he won’t smoke anymore.” Finally, for psychosomatic pain conditions, the healer stated that tobacco cannot directly treat such conditions. It may help indirectly by centering the person’s mind, improving their sleep and appetite; the pain condition itself, however, will necessitate additional treatments. In regards to sleep specifically, the healer adds that topical application of crushed tobacco leaves on the forehead can be very helpful in cases of insomnia or other sleep disturbances.

### Applications in Amazonian Epistemic Health Domains and the Tobacco's Spiritual-Energetic Properties

The healer explains that viewed from the Amazonian medical understanding, tobacco medicine, in addition to the plant’s biochemical action, exerts its therapeutic effects *via* the energetic and spiritual domains. These domains or aspects of the human body are significant for pathogenesis “because illnesses first arise in the spirit-body, then in the energy-body, and only then manifest in the physical body,” he explains. Aiming to further elucidate the energy aspect of the body, he describes that like nerves, the body is permeated by energy channels, which are in direct relationship with the structures of body and mind. In his point of view, the key power of the tobacco plant, thus, lies in its extraordinary capacity to tackle the spiritual-energetic dimension: “There are many excellent medicines, but for energetic problems, tobacco is number one.” He describes that it removes energetic pollutants from the system, thereby increasing the flow of energy in the body, which in turn improves health of body and mind. Furthermore, according to Amazonian epistemologies, there are illnesses that are expressly related to spirits (noncorporeal living beings, which can be health- or sickness-promoting). The tobacco is said to be effective for such illnesses because the plant itself is associated with a powerfully healing spirit, which antagonizes malevolent entities: “With tobacco [these kinds of illnesses] get cured in one go, because demons cannot live where there is tobacco.”

The healer illustrates the interplay between physical, psychological, energetic, and spirit-related factors in the description of the immediate effect of tobacco: “Upon ingestion the tobacco connects to the stomach and, together with its energetic power, connects to the intestines. From the intestines it connects to the brain and nervous system and mobilizes the energy system of the overall body. In the course of this process, vomiting is triggered. [… ] So the person tends to vomit, but in these bouts of vomiting, it is not just vomiting: the patient frees himself from physical ills, but also—for those that are able to understand this—from a lot of bad spirits, and is also cleaning his energetic field. This is what this kind of tobacco that makes one vomit is aimed for. [… ] Once the person has vomited, they have freed their mind, heart, intestines, and overall body. There is an energetic change, the person feels lighter, more joyful, more calm, with a more loving connection towards nature, things like that.”

Consequently, the healer reports the utility of tobacco for a series of health problems which according to Amazonian epistemics are energetic or spiritual in essence and etiology: for instance *brujerías*, *daños, mal del aire*, or *saladera*, which are different classes of illness that have an accumulation of energetic pollutants and/or spirit-related problems in common, and can be treated effectively with tobacco. The person suffering from these conditions may exhibit a specific symptom pattern (e.g., insomnia, diarrhea, paranoia, loss of weight, etc.), but once the tobacco treatment has expelled the unhealthy spirits and energetic toxicities from the person’s body, the symptoms disappear, explains the healer.

Lastly, in the context of the tobacco plant’s spiritual effects, the healer describes a phenomenon he calls “awakening the spirit-body” of a person. He explains that aside from the physical and energy body, every person possesses a spiritual body, which is a natural aspect of the overall human. Most people are however unaware of having this part because it is asleep or sick, he explains. The correct application of tobacco may help this aspect to awaken or recover, he explains, and that “if it has awakened, it functions in one’s dreams; the spirit-body starts to do its work.” The healer strongly emphasizes the personal and interpersonal significance of awakening one’s spirit-body, as it brings about more understanding and care of others, more compassionate and conscious living.

### Contraindications, Interactions, Risks, and Adverse Effects

The healer reports heart diseases, high or low blood pressure, and severely impaired lungs or other vital organs (e.g., cirrhosis of the liver), as well as a “greatly affected nervous system” and the aforementioned mental illnesses as contraindications for tobacco-treatments. Furthermore, the healer explains that tobacco should not be taken during pregnancy or lactation. In terms of drug interactions, the healer mentions antibiotic drugs, which should not be used in the context of a tobacco treatment with a time lapse before and after being necessary. Moreover, recreational drug users may not drink tobacco until they have ceased usage for at least 6 months. Finally, other powerful medicinal plants should not be used in conjunction with a tobacco treatment.

The healer determines the suitability of an individual for tobacco-based treatment *via* the traditional Amazonian diagnostic techniques, involving i.a. the palpation of a patient’s pulse (*pulsar*). Whether the elderly can drink tobacco depends on their overall strength and health condition in the context of their age, states the healer: “If a person, for example, is 70 years old, and has a compromised liver, problems with their prostate, a lot of age-related health issues, then tobacco is not suited for them.” Similarly for children, those below 5 years of age are generally treated *via sopladas* rather than ingestion (minimal age of 6 months for treatment with *soplada*). He explains that regardless of age, for any Amazonian treatment that requires a strict dietary regime like tobacco, a certain level of physical strength is necessary “because a very debilitated body will not withstand the diet.”

In terms of risks of tobacco treatments, the informant highlights first and foremost the proficiency of the healer: “The curandero needs to be a curandero. If a person that is not a curandero serves tobacco, he can kill the patient. [… ] The medicine is very good—that is why it is called ‘medicine’—but used incorrectly it can kill, like any potent medicine.” He explains that if a person serves tobacco to a patient that has a condition for which it is contraindicated, without knowing the diagnostic methods to determine if a patient is apt for the treatment, or if the healer does not understand the effects thoroughly, or does not master the traditional techniques for intervening if needed, the consequences can be fatal. For example, some patients may not vomit for certain reasons, which can be dangerous due to an overheated system; they may want to lie down or faint, and a tabaquero must recognize such cases and be able to intervene with the traditional techniques specifically designed for these situations. Hence, a skilled and experienced healer is absolutely essential for patient safety, the tabaquero emphasizes. Finally, if the mandatory dietary rules of the tobacco treatment are not followed, this may lead to a series of adverse effects ranging from mild to intense.

### Uses of Tobacco Smoke in the Work of a Tabaquero and Amazonian Medicine

“Tobacco in smoked form has energetic purposes,” says the healer, and describes sophisticated techniques, with different areas of application. The smoke is swallowed to the stomach, he points out, not inhaled to the lungs. He highlights that, in order to be able to use tobacco for healing work, an apprenticing tabaquero must first prepare his/her body with the ingestion of medicinal plants in the context of long-term dietary retreats (*dietas*). Furthermore, the trainee must learn the techniques for *soplar* (blowing smoke) and *icarar* (specific chanting for healing purposes). *Soplar* and *icarar* are key for what the healer calls “directing the medicine”: Together they function like a vector, linking the curative forces of the healer with the medicinal properties of the plants and imparting it a direction that guides the therapeutic process. He describes these techniques as an integral part of the therapeutic success: “It is part of the treatment, part of the efficacy of the treatment of the sick person’s body. [… ] If the medicine normally can cure 30%, the curandero by means of his *icaro* and *soplada* adds another 70% of therapeutic force.” Furthermore, a curandero may blow smoke (*soplar*) directly onto a patient’s body to “help a sick person rest and recover their energy.” The smoke applied by the healer in this manner is said to have a protective function “like a shield on your outer body.” He uses a variety of techniques for this: “There are manners of *soplar*, a curandero knows where he needs to blow. There are points on the body where the smoke needs to be blown. [… ] These are energetic points, a bit like acupuncture, for example.” He explains that a healer can treat specific illnesses in this manner, for example *susto*, which is a type of fright or startle response that disrupts the person’s energy system.

Finally, when asked about the difference between smoke that is therapeutic and smoke that is harmful, the healer responds that, first, the tobacco used in industrial cigarettes is poisonous: “The tobacco companies have adulterated the tobacco, adding chemicals. And these chemicals are destructive for the entire human being, from head to toe.” He believes this has been done for financial reasons, so that the people would buy more, like with drugs. “In our case here, the tabaquero has the tobacco plant, and [cured with] alcohol of the sugarcane, it is all natural, no chemicals beyond the natural ones of the plant.” Second, he points to the difference in manner of usage: “The curandero does not smoke for pleasure, there is a purpose. [… ] He smokes when it is necessary.” When asked if the smoking of a tabaquero may eventually have negative health consequences for the tabaquero himself, he explained that a tabaquero undergoes a long-term preparation during his/her apprenticeship for this purpose, preparing the body with specific medicinal plants and diets, so that his body can withstand the work with tobacco. For someone without this preparation, he considers it indeed potentially harmful to use tobacco in that manner.

### Tobacco in Relation to Other Amazonian Medicinal Plants

Tobacco has a unique place in Amazonian medicine, explains the healer, in that it is the one plant used to direct or potentiate the others: “All plant remedies have to be blown with tobacco smoke” before giving them to the patient, says the healer. Because of its healing scope and due to its spiritual-energetic force, he considers tobacco as a sort of royal herb among medicinal plants: “The tobacco’s healing power in the energetic realm is outstanding; I have worked with many other power plants, very good ones, but none of them compared to tobacco in this context.” He also describes that tobacco is closely connected to the “tree medicines” used in the Amazonian tradition, which are also fundamental, he explains, by giving strength and stability to a patient. When asked about the difference between tobacco and ayahuasca (given that the latter is very popular in the world currently), he said that “tobacco centers your mind while ayahuasca produces visions and fantasies.” He considers that without adequate preparation, it is very difficult for most people to differentiate between the reality and the fantasies of their own mind, which is a problem or risk in ayahuasca. He points out that if given to the right patient by an adequately trained healer, both medicines are very valuable. However, he considers that most people using ayahuasca currently are ill-prepared for working with this plant, as this requires an extensive preparation period, which they have not undertaken. “Ayahuasca is very good, but it has to be given by healers who know, who have trees in their body, and have prepared themselves and can direct the work. Not by just anyone. Because today the world is full, there are many who have taken ayahuasca 10 times and are already shamans, and serving ayahuasca—it does not work like that.” He therefore considers there to exist a lot of misguided practice and misunderstandings around this plant at present.

## Discussion

The current study described Amazonian therapeutic uses of tobacco (*N. rustica* L.) in the practice of a *Maestro Tabaquero*, an accomplished Amazonian healer specializing in tobacco-based treatments. The informant’s descriptions revealed refined knowledge on the plant’s therapeutic scope and properties, safety profile, and application techniques. A liquid remedy taken orally was his most commonly used preparation, with reported acute/sub-acute effects involving a pronounced psychoactive component (altered state of consciousness) and physiological response (emesis, intoxication, Spanish: “mareación”). A skilled tabaquero that knows how to diagnose, dose, administer, and intervene in case of adverse effects was described as imperative for safe treatment delivery; tobacco ingestion otherwise may be dangerous. The main indications mentioned by the informant included problems of the mind, of the respiratory system, parasitic illnesses (intestinal/skin), gout, and Amazonian epistemic conditions described as spiritual-energetic in nature.

The stated key indication to treat “problems of the mind,” together with the effects described by our informant as “opening of the psychic-mental,” suggest parallels to psychedelic-assisted therapies, a rapidly growing trend in current psychiatry research (Tupper et al., 2015; Garcia-Romeu et al., 2016; Johnson et al., 2019). Such therapies involve the administration of psychoactive substances (e.g., psilocybin, ayahuasca) to treat mental health problems, with promising results reported for instance for mood, anxiety, addictive, or neurological disorders (Baumeister et al., 2014; Thomas et al., 2017; Palhano-Fontes et al., 2018; Davies and Bhattacharyya, 2019; Butler et al., 2020). Although there is no doubt that tobacco is psychoactive, there has been debate as to the specific class of psychoactive substances it falls. While reported tobacco uses in shamanism involve trances and visions, thus suggesting the plant as a hallucinogen/psychedelic (Janiger and de Rios, 1973; Janiger and de Rios, 1976; Wilbert, 1993), research on nicotine, the tobacco alkaloid mainly studied in this context, considers tobacco a stimulant/depressant (Levin et al., 1998; Picciotto et al., 2002). Neuroscientific studies point to complex actions of nicotine on human cognition, with cognitive-enhancing effects in attentional and memory processes (Valentine and Sofuoglu, 2018). However, nicotine is by no means the only alkaloid or active constituent of the tobacco plant, and tobacco-induced effects might differ from the effects of nicotine alone in significant ways (Hoffmann and Wynder, 1986; Leffingwell, 1999; Charlton, 2004). For instance, aside from nornicotine, anatabine, and anabasine (Benowitz et al., 2009), the β-carbolines harman and norharman which act as monoamine oxidase (MAO) inhibitors have also been reported in tobacco (Janiger and de Rios, 1976; Herraiz and Chaparro, 2005). MAO-inhibiting β-carbolines are currently investigated for their antidepressant effects (Ferraz et al., 2019) and are present also in the Amazonian ayahuasca brew. In fact, their synergistic interaction with other alkaloids of the brew play a key role in the psychopharmacology of ayahuasca (Riba et al., 2003; Palhano-Fontes et al., 2018). Hence, in addition to nicotine, other constituents of tobacco might be critically involved in the psychoactivity of oral tobacco. Overall, and as our findings also imply, the nature of tobacco effects may crucially depend on the plant material used, its preparation and ingredients added, as well as the context of administration (see also concept of “set and setting” in psychoactive plant use, Hartogsohn, 2017). In the case of our informant’s described “strong preparation” (ceremonial or retreat format), broad parallels to other psychedelic-assisted therapies are clearly evident (altered states, spiritual/peak experiences; mental health indications; high levels of anxiety or psychotic features as contraindications), but experimental studies that assess subjective effects directly are needed. Interestingly, the healer’s descriptions of the tobacco effect as centering the mind or aligning are also somewhat evocative of mindfulness concepts. Originally stemming from Buddhist meditative practices, mindfulness-based interventions are common in contemporary psychotherapy to treat depression, chronic pain, or stress reduction (Kabat-Zinn, 2003; Fjorback et al., 2011).

Noteworthy is further the healer’s account of a direct link between intestines and brain involved in the therapeutic mechanism of ingested tobacco, and its beneficial effects on mental parameters related to its action in the gut (expelling of intestinal parasites). The description is in line with the scientific understanding of the gut-brain axis, a close relationship between gut health and brain functioning, whereby alterations in gut microbiota may influence psychological health (Mayer, 2011; Foster and McVey Neufeld, 2013; Mayer et al., 2015). Emerging therapies based on this relatively recent understanding are testing for instance probiotics to treat clinical depression (Nikolova et al., 2019). Beyond its direct action in the neurosystem, nicotine is also known to alter the gut microbiome (Chi et al., 2017) and tobacco’s antiparasitic properties in general are well-documented (Schorderet Weber et al., 2019). At a more basic level, the healer’s description of link between gut and brain may also express the pharmacological process of active tobacco constituents reaching the brain *via* blood pathways after gastrointestinal absorption (oral route). Bearing in mind the aforementioned caveat that the tobacco remedy may not be equated to nicotine alone, the choice of route of administration is interesting, given absorption of nicotine *via* the oral route is subjected to first-pass metabolism, with only a fraction of ingested material reaching the brain (Benowitz et al., 2009; Alkam and Nabeshima, 2019). Pulmonary absorption *via* smoking would be more effective in terms of nicotine levels, which suggests the maximization thereof not to be the main objective or priority of this tobacco therapy, nor the minimization of symptoms of intoxication like vomiting. The latter is in fact a deliberately sought effect of the tobacco remedy: from the Amazonian perspective it is considered an inherent part of the therapeutic mechanism. Lastly, nicotine also has peripheral vasoconstrictive or bronchoconstrictive effects (Alkam and Nabeshima, 2019), which is in line with the healer’s stated contraindications regarding blood pressure and heart or severe lung diseases. It may appear to contrast with his description of increased oxygen flow, however, rather than referring to a direct effect, the latter was stated as an indirect consequence resulting from the tobacco’s removal of phlegm/blockages and subsequent opening of pathways/mind, a process the healer subsumed as “increased oxygen flow.”

Finally, the Amazonian epistemic concepts that play a role in tobacco-based treatments as per our informant are consistent with traditional Amazonian conceptions of healing and sickness in general (e.g., concept of plants with agency, concept of spiritual/energetic factors in pathogenesis and treatment; Luna, 1984; Lenaerts, 2006; Jauregui et al., 2011; Berlowitz et al., 2017). The use of emetic plants and idea of vomiting not as an undesirable side effect, but as key component of therapeutic effectiveness, is characteristic in Peruvian Amazonian medicine (see Sanz-Biset and Canigueral, 2013, for a description of depurative practices in Amazonian medicine) and the concept can be found also in other traditional medicines (e.g., Ayurveda, Bhatted et al., 2011; Vinjamury et al., 2012). The usage of tobacco smoke on the body (*soplada*) might have certain parallels with the practice of moxibustion (application of burning *Artemisia vulgaris*/other herbs on acupoints; Deng and Shen, 2013), the energy channels in the body mentioned by the healer reminiscent of the meridian systems in Asian medicines. The informant used the terms *energy*/*energetic* and *spirit*/*spiritual* in varying contexts in his descriptions. While the former generally denoted subtle vitality forces of the body, the latter was used to describe a noncorporeal (“essence”) aspect of self, or “spirits” in the sense of noncorporeal others which are in interrelationship with humans (see also Amazonian animist ontologies; Viveiros de Castro, 1998; Rosengren, 2006; Santos-Granero, 2006). The tabaquero’s description of etiology of diseases, which “first arise in the spirit-body, then in the energy-body, and only then manifest in the physical body,” is interesting in conjunction with his stated mode of action of tobacco in terms of its effectiveness in the spiritual/energetic domain: It implies that from the Amazonian epistemic perspective, tobacco has the capacity to intervene in the process of pathogenesis at a level more fundamental than the physical. This may provide the rationale for the healer’s declared supremacy of the plant (“royal plant”), which is in line with ethnographic accounts of tobacco’s central role in Amazonian healing (e.g., “father of all plants”) and in the etymology of the healer-term in indigenous Amazonian languages (e.g., “he who eats tobacco”), as outlined in the introductory section (Shepard, 1998; Rosengren, 2006; Barbira-Freedman, 2010; Thomas et al., 2011; Russell and Rahman, 2015; Barbira-Freedman, 2015). It also is in line with the prominence of tobacco uses in other Amerindian cultures across the Americas (Wilbert, 1993; Groark, 2010; Russell and Rahman, 2015; Rodríguez Arce and Cerdas, 2019), where it has long been a highly esteemed plant, its consciousness-altering effects harnessed by shamans for divination, sorcery, healing, or spiritual purposes since millennia (Janiger and de Rios, 1973; Zethelius and Balick, 1982; Wilbert, 1991; Echeverría and Niemeyer, 2013; Tushingham et al., 2018). Finally, also consistent with our findings are reports of indigenous tobacco uses in horticulture (as an insecticide) and in veterinary medicine, or to improve dogs’ hunting skills (Wilbert, 1993; Bennett and Alarcón, 2015).

The healer reports using tobacco leaves that have been cured with sugarcane alcohol, a procedure that is aimed to obtain a tobacco of high medicinal value or efficiency that can be conserved in the hot/damp climatic conditions of the Amazon. This is in contrast to tobacco cured with sweet sugarcane derivatives in other contexts (e.g., usage of molasses in the Indian sub-continent), that flavor the tobacco to enhance the pleasurable experience of smoking (Talhout et al., 2006).

This work has several limitations. We opted for a single-respondent design, conducting repeated in-depth interview sessions with a carefully selected informant. This had advantages from a clinical point of view (generating data of great detail and depth) but came at the cost of generalizability across healers or cultures. As a consequence, our findings give insight into the practice of a highly accomplished expert in this field, but a broader, cross-ethnic interview study would be needed to know if they are representative of Peruvian-Amazonian tobacco uses in a more general sense. More importantly from a clinical perspective, the qualitative expert interview approach (Bogner and Menz, 2009) we used should be complemented with case reports and a direct quantitative assessment of patients, as a next step. To our knowledge, this is the first study employing a transdisciplinary clinical focus to examine therapeutic applications of tobacco by an Amazonian tabaquero. Our findings significantly contribute to the growing research literature on Amazonian medicine and on emergent psychedelic-assisted therapies and could, in the long-term, open new treatment avenues in several domains. Forthcoming studies should assess toxicity/safety and clinical outcomes of patients receiving Amazonian tobacco-based treatment.

## Data Availability Statement

The datasets presented in this article are not readily available because: Protection of traditional medical knowledge of Amazonian healers. Requests to access the datasets should be directed to ilana.berlowitz@unifr.ch.

## Ethics Statement

Written informed consent was obtained from the individual(s) for the publication of any potentially identifiable images or data included in this article.

## Author Contributions

IB and CM-S were responsible for conception and design of the study and acquired funding for this project. EGT offered guidance to the design and provided access to knowledge on tobacco-based treatments and infrastructure for fieldwork. IB wrote the first draft of the manuscript, managed data collection and analysis, and submitted the manuscript. CM-S critically revised the manuscript and offered advice from neuropsychology. CM provided critical insight from the biomedical perspective, assisted the submission process and manuscript revision. UW offered critical advice from the perspective of complementary and integrative medicine to the study, manuscript revision and advised the construction of the interview schedule. HW advised the study from the biological perspective. IB prepared the interview schedule, and all authors contributed to its revision and finalization. All authors contributed to the article and approved the submitted version.

## Funding

This research was funded by the Swiss National Science Foundation as part of a Spark grant (SNF-190428). The University of Fribourg Research Pool and the Inger-Salling Prize supported this work *via* contributions to grant writing.

## Conflict of Interest

The authors declare that the research was conducted in the absence of any commercial or financial relationships that could be construed as a potential conflict of interest.
